# The use of precision medicine for asthma

**DOI:** 10.3389/fmed.2025.1743829

**Published:** 2026-01-13

**Authors:** Lalitha Menon, Alice Crawford, Zoe Castillo, Christine Jenkins, John D. Blakey

**Affiliations:** 1 Department of Respiratory Medicine, Sir Charles Gairdner Hospital, Perth, WA, Australia; 2 Institute for Respiratory Health, Perth, WA, Australia; 3 Respiratory Group, The George Institute for Global Health, Sydney, NSW, Australia; 4 Respiratory Medicine, University of New South Wales Sydney, Sydney, NSW, Australia; 5 Curtin Medical School, Curtin University, Perth, WA, Australia

**Keywords:** asthma, population health, precision medicine, primary care, specialist care

## Introduction

The introduction of articles about asthma often begin by describing the condition as affecting over 300 million people globally (1). This is usually followed by a statement regarding the poor outcomes observed for people with asthma. Indeed, asthma leads to a substantial amount of preventable physical and psychological morbidity (2), unscheduled healthcare use and mortality (3). It is clearly also a major health economic issue, with billions of dollars consumed in direct costs and loss of productivity (4). It is not surprising that these facts are used as support for the development of precision medicine approaches. In this article, we consider where precision medicine could and should be reasonably deployed to improve asthma outcomes. We consider what benefits could be potentially realized through newer precision medicine approaches, and put this in the context of gains that can be made using existing approaches and treatments for asthma.

## A global disease

Asthma can affect people of any age, in any location, and of any heritage. Although an elevated prevalence of asthma is notable in higher income countries such as the United States, Australia and the United Kingdom (5), the majority of people with asthma live in lower and middle income countries. These countries also face the greatest burden of uncontrolled asthma and preventable deaths from asthma (6). The overwhelming issue faced in such countries is appropriate access to healthcare for diagnosis and the availability and affordability of standard preventative therapy, and primarily not a lack of highly specialist management of asthma and related conditions. That said, matching the appropriate treatment to the appropriate patient has tremendous potential to make more efficient use of medications. Improved asthma outcomes will also reduce strain on overstretched acute healthcare systems. The pursuit of affordable and meaningful biomarkers that are robust to lower income environments is therefore an essential long-term endeavor if we are to improve global asthma outcomes.

In higher income countries, there is an equally concerning but near opposite problem. Medical waste in the form of overtreatment and unnecessary investigations is a serious challenge, with reports that approximately 30% of healthcare is unhelpful and 10% of healthcare is harmful (7). It has been apparent for some time that asthma is over-diagnosed; literature suggests a third of people treated for asthma have no objective evidence of the condition when tested (8). The problem of overdiagnosis in adults is in part due to an assumption that a history of wheezing in childhood means that recurrent symptoms in adults are most likely due to asthma. It is essential that adults are reassessed thoroughly and diagnostic tests are employed to confirm or question the reappearance of asthma-like symptoms.

The lack of concordance with asthma diagnosis is also reflected in treatment regimens used in higher income countries, which are not always evidenced based. Many people with airways diseases are prescribed treatments that do not follow guidelines (9–11), and less than 10% of people are concordant with preventative inhaled therapy when this is assessed objectively using a smart inhaler (12). Infrequent use of basic inhaled corticosteroid-containing preventative therapy and over-reliance on short-acting beta-agonist drugs are associated with poor outcomes (13). Asthma maintenance therapy has evolved significantly over the last decade, but uptake has been slow. Therefore, at a population level there is a pressing need to get the basics of asthma management in place before considering precision medicine.

## Precision medicine in primary care?

Most people with asthma are managed in a primary care setting. Core elements of an asthma assessment include an appraisal of current symptom control and an estimation of the risk of future asthma attacks. The role of primary care is to deliver personalized, patient centered medicine. In essence, precision medicine is a component of personalized medicine. It involves tailored treatment based on an individual's genetic, environmental and lifestyle factors. When factors such as exacerbation history, allergy history and the presence of biomarkers such as blood eosinophil count, FeNO or serum IgE are clustered together into recognized patterns, they are often termed phenotypes. When there is a clear biological pathway between pathology, biomarker and clinical presentation the term “endotype” is often applied. Identifying asthmatic phenotypes and endotypes facilitate the construction of targeted, bespoke management to optimize outcomes. An example of personalized medicine in asthma involves selecting the optimal inhaled corticosteroid for the patient. Though inhaler devices are equally efficacious in drug delivery (14), real world problems of poor patient dexterity, adherence to twice daily vs. daily dosing and poor inspiratory strength along with infrequent review of inhaler technique remain barriers to optimal asthma control (15). With the aid of primary care nurses (16), spacers and online asthma tools, finding the correct inhaler is a simple and effective way of personalized medicine being used to prevent asthma attacks.

It is readily appreciated that asthma attacks are not random occurrences and many studies have sought to quantify the predictive ability of clinical parameters for future asthma attacks (17). This has been demonstrated successfully in large primary care database studies (18). The addition of biomarker measurement to clinical parameters such as exacerbation history, disease severity and symptoms (19) in primary care asthma risk prediction is currently of uncertain benefit, even though high variability in eosinophil counts is associated with higher risk of severe attacks (20). In a study of children visiting the emergency department in a lower income area of Ecuador where inhaled corticosteroids are not routinely used, the addition of exhaled nitric oxide and blood or nasal eosinophil counts did not add to a clinical model predicting future emergency visits (21). Studies that have added biomarkers into a predictive model show some promise, however, they have been derived from the control arms of randomized control trials so their generalizability remains unclear (22).

**Figure 1:**
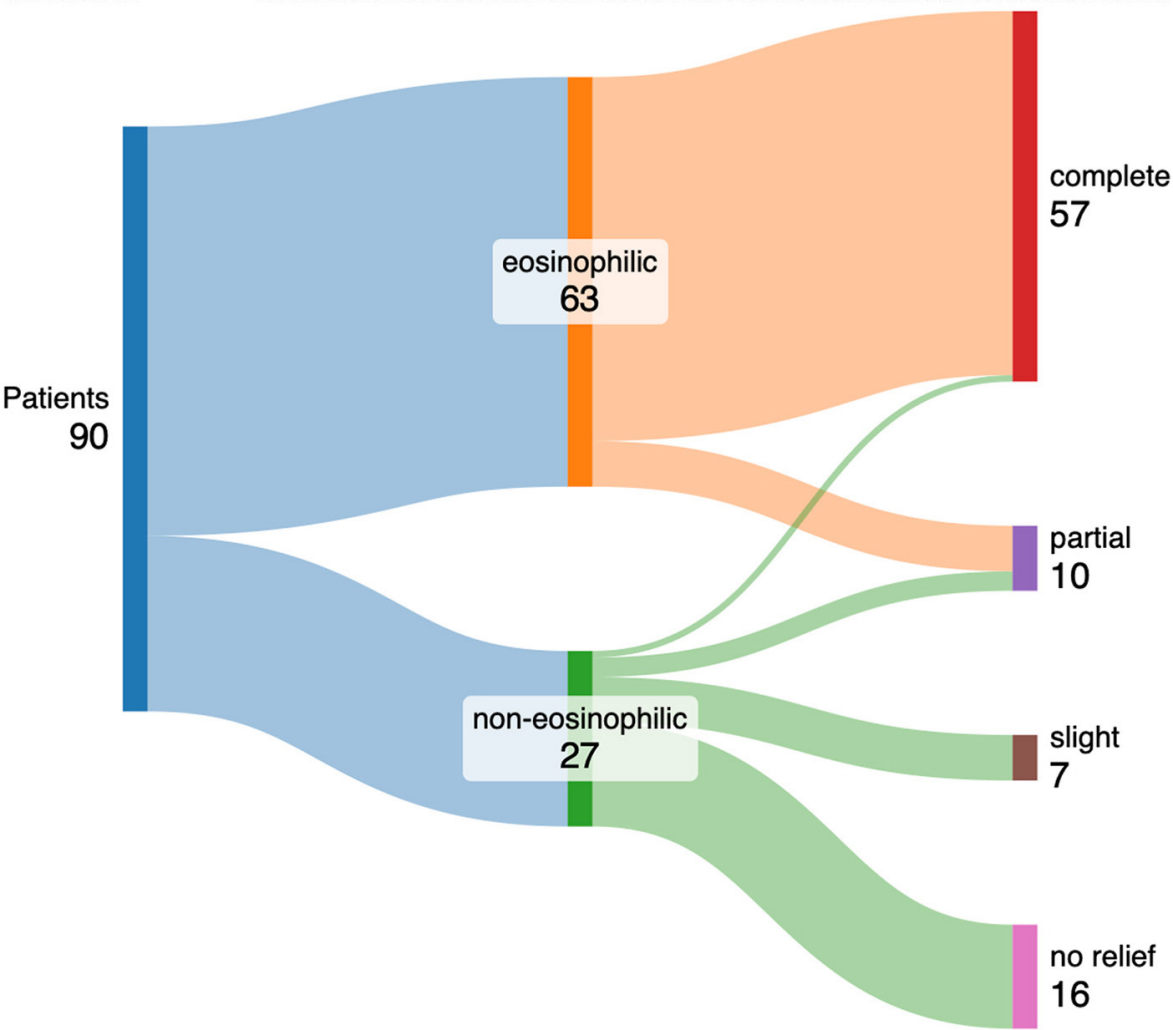
Shows Sankey diagram representing the outcomes of patients with asthma who were given oral corticosteroids, categorized by their sputum eosinophil count.

So how can precision medicine be implemented in primary care? One way would be to use biomarkers to guide oral corticosteroid (OCS) prescribing for asthma exacerbations. OCS stewardship is a pressing issue in respiratory medicine. The serious potential harms of OCS have been known since the mid-1950s (23). More recently, we have come to understand the adverse effects accrue from a modest cumulative lifetime exposure to OCS, and that most people with asthma appear to exceed this threshold (24). However, we have also learnt that OCS are not beneficial to all patients with asthma (25), and that biomarker measurement successfully guides appropriate use. In his seminal paper, Harry Morrow Brown showed that the presence of eosinophils in sputum was predictive of OCS response in asthma (see Figure 1) (26). We have since seen that blood eosinophils can be successfully employed as a predictor of the outcome of OCS therapy for asthma and COPD (27). By using this precision medicine technique, we can avoid automatic OCS prescription for all attacks simply by checking an eosinophil count, and minimize OCS toxicity in asthma patients in primary care setting.

Overall, it does not appear there is a need for additional precision medicine biomarkers to answer the question “should this patient be referred for specialist therapy?”. Rather than using a blood eosinophil count or exhaled nitric oxide numerical value, we should instead focus on recurrent exacerbations, difficulty achieving control and cumulative OCS dosing to prompt referral for specialist review. The main issue is once again concordance with guidelines: less than a quarter of individuals that meet the criteria for specialist review are referred, and there is often a long waiting period before specialist review (28). Work is underway by several groups to improve this situation, including the Future of Asthma project which advocates for newer ways of delivering current effective interventions (29).

## Precision medicine in specialist care?

Once the decision has been made that something must be done, the question of “what is that something?” is more challenging. Precision medicine appears a more natural fit for specialist care, given that the main function of a specialist asthma service is to expand on management that cannot be effectively delivered in primary care. Specialist services have the luxury of time with the patient, in addition to access to multidisciplinary care which allows for a greater collective expertise when treating asthma.

It has been shown that the great majority of individuals referred to specialist asthma services do not receive “specialist medicines,” but rather have greater support with other issues such as medication/inhaler technique and adherence, smoking cessation and weight loss (30). Conditions that mimic or exacerbate asthma symptoms such as dysfunctional breathing or inducible laryngeal obstruction are commonly identified. A multidisciplinary, multi-dimensional approach to asthma management has been shown to improve asthma symptoms and quality of life and reduce exacerbations and oral corticosteroid exposure (31). These gains are often made through the identification and treatment of common comorbidities rather than targeted therapies. Multiple comorbidities are not only seen in those attending specialist services, but in individuals in the community with poor control (32). The treatable traits approach to asthma (33) is perfect example of personalized medicine utilized in specialist care (and possibly primary care), as it identifies and manages pulmonary, extrapulmonary and lifestyle specific factors that lead to poor asthma control.

The past decade has seen increasing use of monoclonal antibody therapy for asthma, first with anti-IgE, then with anti-IL5, anti-IL5RA, anti-IL4, and IL13 and anti-TSLP. This has led to significant academic interest in using biomarkers to select the optimal asthma antibody treatment for an individual. The asthma exacerbation reduction seen with monoclonal antibody therapy is marked, and significantly greater than the difference between them. For example, the anti-IL5 antibody mepolizumab bestows the same protective effect on exacerbations regardless of serum IgE level (34). Furthermore, a recent randomized trial showed individuals with severe allergic asthma meeting the criteria for omalizumab did just as well if given mepolizumab instead (35). In the context of our current therapeutic armamentarium, the benefits of being comprehensively assessed by a specialist team and started on a monoclonal antibody outweigh the additional benefit that one drug may offer over another.

## Unmet need and precision medicine

Although there are major potential gains and benefits to be realized with improved use of current asthma treatments, this should not detract from the parallel problem of there being significant harms to be avoided. We see type 2 (Th2) inflammatory markers like blood eosinophil counts or exhaled nitric oxide as a success in introducing precision medicine into asthma care. Several studies have shown that the risk of exacerbation and lung damage rise with increasing eosinophil count, and that treatments that address Th2 inflammation can ameliorate this risk. However, people with asthma who are adherent to regular monoclonal antibody therapy continue to experience exacerbations. This situation has important lessons for future research into the use of biomarkers for targeted therapy. Firstly, Th2 biomarkers are not stable over time. If individuals are retested at intervals, a substantial proportion change their risk-profile from Th2-high to Th2-low or vice versa (36, 37). Secondly, in individuals selected for trials on the basis of an eosinophilic phenotype, and who have their eosinophils abolished by the monoclonal antibody benralizumab, exacerbations continue to occur across all subpopulations (38). Thirdly, exacerbations occur in individuals with asthma who persistently have low blood eosinophils and FeNO, seemingly at approximately the same rate as those who do not (39). These findings indicate that there are clearly other mechanisms and pathways at work that lead to asthma exacerbations and that targeting Th2 biomarkers alone are not the answer to true asthma control.

With the increasing understanding of the underlying biology of asthma, and a variety of newer medications coming to market, we should look to the future of asthma management with optimism. We already have highly effective inhaled medications and new strategies that are tailored to patient preferences and behavior (AIR and MART). Our clinical responsibility is to focus on making the correct diagnosis and managing each patient with the medicines that suit them best, and that they will take in the long term to avoid unnecessary harms. As expected, newer treatments are likely to be costly, and it is therefore unlikely that healthcare systems will be able to offer these therapies at scale or be able to offer multiple highly specialized therapies to one individual. The question of which individuals are most likely to benefit (and least likely to be harmed) by treatment will therefore become the main dilemma of specialist management. Although this financial reality will be a major driver for precision medicine in asthma in the coming decade, we contend that medical treatment tailored to an individual's phenotype is also achievable for many or even most patients, without these expensive interventions.

People can acquire asthma at any point in their life, and for the great majority of individuals, a cause cannot be identified. The lingering thought persists, though, that if something switched asthma on, it should be possible to switch it off. Some observations give hope this is possible. Spontaneous resolution of asthma is an uncommon but objective phenomenon, and the use of monoclonal antibodies has achieved disease remission in a minority of individuals with previously severe uncontrolled asthma, while continuing treatment. The identification of subsets of asthma that might be amenable to a curative treatment has for some time been beyond our reach. However, with greater bio-banked resources and newer scientific techniques, ambitious projects such as CURE Asthma are beginning the process of working toward cures (40). The goal of precision medicine in asthma over the next 10 to 20 years may not be simply in treatment selection, but to identify particular causative pathways and deliver therapy to normalize aberrant signaling, resulting in asthma cure. An essential aspect of this should be to reduce the inequity that currently exists so that people with asthma in high and low income settings have a hope, not only of fewer attacks and better control, but also of cure.

## Summary

**Figure 2:**
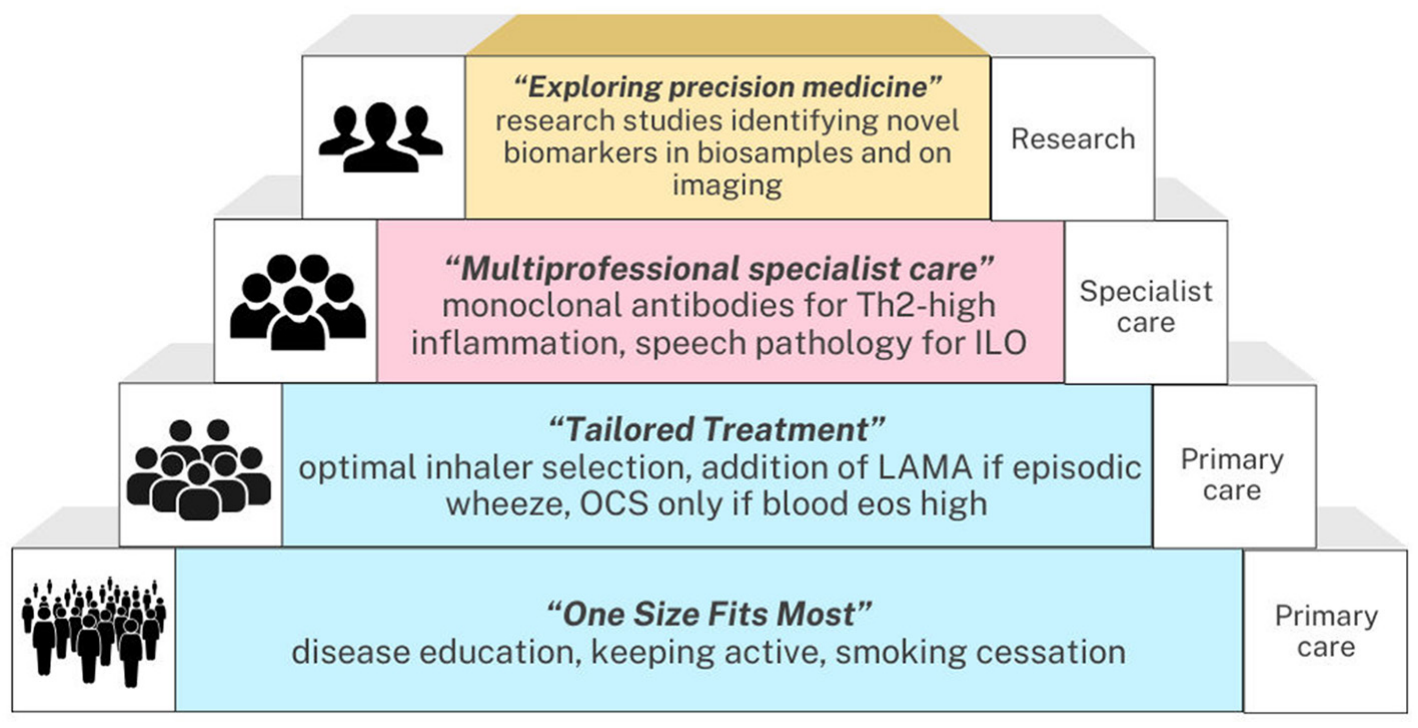
Concept diagram of overview of the relationship between precision medicine and other aspects of asthma care.

Precision medicine for asthma will look different in the primary care setting compared to specialist care (see Figure 2), however our current ability to assess who is at risk of asthma exacerbations and to provide treatments to ameliorate that risk in both domains of healthcare remains significant. With an increasing array of targeted asthma medications becoming available, the utilization of newer therapies where there is the greatest therapeutic index will be essential to maximizing patient outcomes, reducing patient harm and minimizing medical waste.
